# Maximizing the detection rate of hypoglycemia among preterm neonates admitted in Neonatal intensive care unit in Ethiopia, 2021

**DOI:** 10.1038/s41598-023-29112-y

**Published:** 2023-02-09

**Authors:** Ermias Sisay Chanie, Sahlu Mitku Shiferaw, Dejen Getaneh Feleke, Berihun Bantie, Natnael Moges, Sheganew Feten Tasew, Tikuneh Yetneberk Alemayehu, Assefa Agegnehu Teshome, Gebrie Kassaw Yirga, Ayenew Berhan, Abraham Tsedalu Amare, Mebratu Libanos, Wondosen Addis Emrie, Sewnet Sisay Chanie

**Affiliations:** 1 Debre Tabor University, Debre Tabor, Ethiopia; 2 Debre Tabor Health Science College, Debre Tabor, Ethiopia; 3 Debre Birhan University, Debre Birhan, Ethiopia

**Keywords:** Endocrinology, Health care, Medical research, Risk factors

## Abstract

The burden of hypoglycemia is high in resource limited countries, such as Ethiopia. However, there are no sufficient studies conducted in Ethiopia in general and in the study setting in particular in the previous era. Hence, this study aims to assess the proportion of hypoglycemia and associated factors among preterm neonates admitted to the neonatal intensive care unit at Debre Tabor Comprehensive Specialized Hospital, Ethiopia, in 2021. A hospital-based cross-sectional study was conducted from October 1 to December 30, 2021, at Debre Tabor Comprehensive Specialized Hospital in the neonatal intensive care unit ward. The data was entered in Epi-info 7 and exported to STATA version 14. A binary and multivariable logistic regression was computed at 95% confidence interval (CI). During bivariable analysis, variables having a *p*-value of less than 0.25 were chosen for multivariable logistic regression analysis, and variables having a *p*-value of less than 0.05 in multivariable analysis, were significant associations with the dependent variable. The study included 267 preterm neonates, and 23.59% (95% CI 18.9–29.1) were develop hypoglycemia. Moreover, 49 (18.35%) preterm neonates died during the study period. In this study, preterm neonates with hypothermia [Adjusted Odds Ratio (AOR = 4.5; 95 CI 3.4, 7.2)], birth asphyxia (AOR = 5.1; 95 CI 3.9, 27.1), seizure (AOR = 4.7; 95 CI 2.8, 17.8), and also preterm neonates born from diabetic mothers (AOR = 6.7; 95 CI 3.3, 27.2) were significantly associated with the occurrence of hypoglycemia in the neonatal intensive care. The proportion of hypoglycemia and associated factors among preterm neonates admitted to the neonatal intensive care unit at Debre Tabor Comprehensive Specialized Hospital was found to be high. The associated factors for the occurrence of hypoglycemia were discovered to be neonates with hypothermia, birth asphyxia, seizure, and neonates born with a diabetes mother. Thus, recognizing and treating the above associated factors is essential to preventing, and controlling hypoglycemia.

## Introduction

Neonatal hypoglycemia is characterized by a lower-than-normal blood glucose level^1^, which is a plasma glucose level of less than 30 mg/dL in the first 24 h of birth and less than 45 mg/dL in the last 24 h of life^2^. Prematurity, small for gestational age, prenatal hypoxia, and neonates delivered to diabetic moms are all at risk for neonatal hypoglycemia^3,4^.

Preterm neonates are particularly susceptible to developing hypoglycemia and its associated complications because of their low glycogen and fat reserves, inability to produce new glucose through gluconeogenesis pathways, higher metabolic demands brought on by their relatively larger brains, and inability to mount a counter-regulatory response to hypoglycemia^5,6^.

In many circumstances, preventing hypoglycemia is the most critical event during the prenatal to neonatal transition period^7^. However, hypoglycemia is the most prevalent metabolic abnormality in the newborn period^4,8^, which is linked to increased neonatal morbidity and mortality^3^.

If left untreated, persistent or recurrent hypoglycemia can have serious neurologic and developmental consequences^9,10^. Moreover, chronic hypoglycemia causes long-term neurodevelopmental problems, cerebral palsy, and mortality in the neonatal era^9,11^. Hence, early detection of recurrent or chronic hypoglycemia is crucial to improving the survival of neonates^9,12–14^.

The challenges to diagnosis and preventing neonatal hypoglycemia are due to their numerous and unclear symptoms^15–17^, which cause common neonatal complications, such as severe malaria, bacterial sepsis, severe malnutrition, and newborn sickness^7,18,19^. Hence, healthcare providers must be on the lookout for all possible indicators of neonatal hypoglycemia with risk factors to maximize detection and treatment of neonates with hypoglycemia^20^.

Although the prevalence of neonatal hypoglycemia varies depending on the definition, demographic, feeding method, timing, and type of glucose test used^2,10^. The overall incidence of hypoglycemia in neonates ranges from 1.3 to 3 per 1000 live births^10^, with risk groups ranging from 30 to 60%^8,11^.

In Ethiopia, the burden of neonatal hypoglycemia is very high^8,10^, and it is also interlinked with numerous neonatal complications and mortality^3,9^. In this regard, screening and managing neonatal hypoglycemia is a challenge for healthcare providers in the neonatal intensive care unit^11,17,19^. Thus, data is essential to recognizing and managing hypoglycemia in preterm neonates. However, there are no sufficient studies conducted in Ethiopia in general and in the study setting in particular in the previous era. Hence, this study aims to assess the rate of hypoglycemia and associated factors among preterm neonates admitted to the Neonatal Intensive Care Unit at Debre Tabor Comprehensive Specialized Hospital, Northcentral, Ethiopia, in 2021.

## Methods and materials

### Study setting and study design

A hospital-based cross-sectional study was conducted from October 1 to December 30, 2021, at Debre Tabor Comprehensive Specialized Hospital in the Neonatal Intensive Care Unit ward. The hospital is located 670 km from Addis Ababa, the capital city of Ethiopia^21^.

### Study participants

All preterm neonates admitted to the neonatal intensive care unit from October 1 to December 30, 2021, at Debre Tabor Comprehensive Specialized Hospital were eligible for the study. Preterm neonates admitted without mothers or caregivers were excluded in the study. Sampling size and.

### Sampling procedure

The sample size was determined using the single population proportion formula by considering the following assumptions: 95% confidence interval, 5% margin of error (d), and taking the p value from the previous study conducted in St. Paul’s Hospital Millennium Medical College Neonatal Intensive Care Unit, Ethiopia^8^.

$$n = \frac{{\left( {z_{a/2} } \right)^{2} \times p\left( {1 - p} \right)}}{{d^{2} }}n = \frac{{\left( {1.96} \right)^{2} \times 0.25\left( {1 - 0.75} \right)}}{{0.05^{2} }} = 289$$

Therefore, the final sample size after adding 5% was 304. However, there were 279 preterm neonates admitted and meeting the inclusion criteria at Debre Tabor Comprehensive Specialized Hospital between October 1 and December 30, 2021.Hence, we included all preterm neonates admitted to the neonatal intensive care unit at Debre Tabor Comprehensive Specialized Hospital during the study period.

### Data collection tools and procedures

The data was collected by interviewing mothers and caregivers. Besides, the clinically relevant information was obtained from the medical records of the neonate. The abstraction tool contained socio-demographic, obstetrics, and preterm neonate-related characteristics that enabled the evaluation of the outcome variable. At first, the abstraction tool was developed in English and translated into the local language, Amharic, and then back to English to keep consistency.

### Operational definitions

Hypoglycemia is defined as a plasma glucose level of less than 47 mg/dL during the study period^22^.

Hypothermia is defined as an abnormal thermal state in which the newborn's body temperature drops below 36.5 °C^23^.

Preterm refers to a baby born before 37 weeks of pregnancy have been completed^24^.

Birth asphyxia is defined as a profound metabolic or mixed acidemia, the persistence of an Apgar score of 0–3 for longer than 5 min^25^.

Neonatal sepsis is defined as a clinical sign with the presence of risk factors, lab tests, or confirmed by blood culture^25^.

### Statistical analysis

The data was entered in Epi-info 7 and exported to STATA version 14. Descriptive statistics such as frequencies, proportions, and cross tabulation were employed. The crude odds ratio (COR) and adjusted odds ratio (AOR) were calculated at 95 percent confidence intervals (CI) for binary and multivariable logistic regression to examine the strength of association between the outcome variable and independent factors. During bivariable analysis, variables having a *p*- value of less than 0.25 were chosen for multivariable logistic regression analysis, and variables having a *p*-value of less than 0.05 in multivariable analysis had significant associations with the dependent variable.

### Ethical approval

Ethical clearance was obtained from the Ethics Review Committee of Debre Tabor University, Ethiopia with Ref NO/HP/713/01/2021 G.C. And then, the official letter was obtained from Debre Tabor Comprehensive Specialized Hospital for permission. All methods were carried out in accordance with relevant guidelines and regulations. The informed consent was obtained from all subjects and/or their legal guardian(s). The names and medical record identification numbers were not collected to keep the study participants' privacy.

## Results

### Socio-demographic and obstetric characteristics of the mother and preterm neonates

Out of 279 study participants, 267 were included in the study, with a completion rate of 95.7%. The mean age with standard deviation (SD) of the mother was 28.7 (6.9) years old. Nearly half (50.19%) of mothers were in the age group of 20–34 years. The majority (74.16% and 85.0%) of mothers were from urban areas and had spontaneous vaginal delivery.

From the total of mothers, 58.80%, 13.48%, and 15.73% of mothers had RH (Rhesus) + ve, Antepartum Hemorrhage (APH), and Premature rupture of membranes (PROM) respectively. Moreover, 13.48% and 18.35% of mothers had diabetes mellitus (DM), respectively. Of the total mothers included in the study, 83.15% of them had ante-natal care (ANC). Of these, 74.91% of mothers had had at least four ANC follow-ups.

From the total of preterm neonates included in the study, 52.43% and 76.78% were male and had a birth weight of >  = 1500 g, respectively, during the study period. The majority of preterm neonates, 85.02%, were initiated by breastfeeding for one hour, and 83.52% had a 5th minute Appearance, Pulse, Grimace, Activity, and Respiration (APGAR) score of greater than 7. During the study period, 26.97%, 17.60%, 10.86%, and 10.11% of preterm neonates had hypothermia, sepsis, birth asphyxia, and seizures, respectively (Table 1).

**Table 1:** Sociodemographic and obstetric characteristics of mothers and preterm neonates at Debre Tabor Comprehensive Specialized Hospital, Ethiopia, in 2021.

Age of mother	20 yrs	23	8.61
	20–34 yrs	134	50.19
	> 34 yrs	110	41.20
Residence	Rural	69	25.84
	Urban	198	74.16
Onset of labor	Spontaneous	227	85.02
	Induced	40	14.98
Maternal RH-factor	+ ve	157	58.80
	− ve	110	41.20
Had APH	Yes	36	13.48
	No	231	86.52
Had ANC follow-up	No	45	16.85
	Yes	222	83.15
Number of ANC visit	< 4	67	25.09
	> = 4	200	74.91
PROM	Yes	42	15.73
	No	225	84.27
Maternal diabetes	Yes	36	13.48
	No	231	86.52
Preeclampsia	Yes	49	18.35
	No	218	81.65
Sex of neonates	Male	140	52.43
	Female	127	47.57
Birth weight	< 1500 g	62	23.22
	> = 1500 g	205	76.78
Initiation of breastfeeding	< 1 h	227	85.02
	> = 1 h	40	14.98
5th min APGAR score	< 7	44	16.48
	> = 7	223	83.52
Hypothermia	Yes	72	26.97
	No	195	73.03
Sepsis	Yes	47	17.60
	No	220	82.40
Birth asphyxia	Yes	29	10.86
	No	238	89.14
Seizure	Yes	27	10.11
	No	240	89.89
Neonate status (live)	Yes	49	18.35
	No	241	81.65

APH, Antepartum Hemorrhage; ANC, Ante-natal care; APGAR, Appearance, Pulse, Grimace, Activity, and Respiration; DM, Diabetes mellitus; PROM, Premature rupture of membranes; RH, Rhesus.

### The proportion of hypoglycemia

In this study, the proportion of hypoglycemia was found to be 23.59% (95% CI 18.9–29.1). Moreover, 49 (18.35%) of preterm neonates died during the study period.

### Associated factors of hypoglycemia

Both bi-variable and multivariable logistic regression was computed. In binary logistic regression, variable including PROM, maternal diabetes, preeclampsia, sex of neonates, birth weight, initiation of breastfeeding, 5th min APGAR score, hypothermia, sepsis, birth asphyxia, and seizure were having a *p*- value of less than 0.25 at 95% CI with hypoglycemia. Whereas, in multivariable analysis, variable including maternal diabetes, hypothermia, birth asphyxia, and seizure were having a *p*-value of less than 0.05 at 95% CI, which had statistically significant associations with hypoglycemia.

In this study, preterm neonates with hypothermia were 4.5 times more likely to develop hypoglycemia than preterm neonates without hypothermia (AOR = 4.5; 95 CI 3.4, 7.2). Likewise, preterm neonates with birth asphyxia were 5.1 times more likely to develop hypoglycemia than preterm neonates without birth asphyxia (AOR = 5.1; 95 CI 3.9, 27.1). Preterm neonates who had seizure were 4.7 times more likely to develop hypoglycemia than preterm neonates who had no seizure (AOR = 4.7; 95 CI 2.8, 17.8). Preterm neonates born of diabetic mothers were 6.7 times more likely to develop hypoglycemia than preterm neonates born without a diabetic mother (AOR = 6.7; 95 CI 3.3, 27.2) (Table 2).

**Table 2:** Associated factors of hypoglycemia at Debre Tabor Comprehensive Specialized Hospital, Ethiopia, in 2021.

Variable	Hypoglycemia		COR (95% CI)	*P*-value	AOR (95% CI)	*P*-value
	No	Yes				
Age of mother						
20 yrs	18	5	0.9 (0.3–2.7)	0.84		
20–34 yrs	102	32	1.0 (0.6–1.8)	0.96		
> 34yrs	84	26	Ref	–		
Residence						
Rural	55	14	0.8 (0.4–1.5)	0.45		
Urban	149	49	Ref	–		
Onset of labor						
Spontaneous	176	51	Ref	–		
Induced	28	12	0.7 (0.3–1.4)	0.30		
Maternal RH-factor						
+ ve	130	27	Ref	–	Ref	
− ve	74	36	2.3 (1.3–4.2)	0.00	2.3 (0.9–5.8)	0.08
Had APH						
Yes	27	9	1.1 (0.5–2.6)	0.83		
No	177	54	Ref	–		
Had ANC follow-up						
No	32	13	1.4 (0.7–2.9)	0.36		
Yes	172	50	Ref	–		
Number of ANC visit						
< 4	54	13	0.7 (0.4–1.4	0.35		
> = 4	150	50	Ref	–		
PROM						
No	176	51	0.5 (0.2–1.2)	0.13	0.4 (0.08–1.6)	0.179
Yes	2	12	Ref	–	Ref	–
Maternal diabetes						
Yes	9	27	16.2 (7.0–36.1)	0.00	6.7 (3.3–27.2)	0.000
No	195	36	Ref	–	Ref	–
Preeclampsia						
Yes	24	25	4.9 (2.6–9.5)	0.00	2.9 (0.9–8.9)	0.053
No	180	38	Ref	–	Ref	–
Sex of neonates						
Male	101	39	1.7 (0.9–3.0)	0.09	2.0 (0.8–5.2)	0.17
Female	103	24	Ref	–	Ref	–
Birth weight						
< 1500gm	51	11	0.7 (0.4–1.4)	0.22	0.7 (0.5–1.1)	0.06
> = 1500gm	153	52	Ref	–	Ref	–
Initiation of breastfeeding						
< 1 h	172	55	Ref	–		
> = 1 h	32	8	0.8 (0.3–1.8)	0.56		
5th min APGAR score						
< 7	33	11	1.1 (0.6–2.3)	0.81		
> = 7	171	52	Ref	–		
Hypothermia						
Yes	33	39	8.4 (4.5–15.8)	0.00	4.5 (3.4–7.2)	0.000
No	171	24	Ref	–	Ref	–
Sepsis						
Yes	33	14	1.5 (0.7–2.9)	0.273		
No	171	49	Ref	–	Ref	–
Birth asphyxia						
Yes	4	25	32.9 (10.8-	0.00	5.1 (3.9–27.1)	0.000
No	200	38	Ref	–	Ref	–
Seizure						
Yes	3	24	5.8 (4.3–13.6)	0.00	4.7 (2.8–17.8)	0.000
No	201	39	Ref	–	Ref	–

APH, Antepartum Hemorrhage; ANC, Ante-natal care; APGAR, Appearance, Pulse, Grimace, Activity, and Respiration; DM, Diabetes mellitus; PROM, Premature rupture of membranes, RH, Rhesus.

## Discussion

The proportion of hypoglycemia and associated factors among preterm neonates admitted to the neonatal Intensive Care Unit at Debre Tabor Comprehensive Specialized Hospital, Northcentral, Ethiopia was found to be 23.59% (95% CI 18.9–29.1). Furthermore, hypothermia, birth asphyxia, seizure, and neonates born to diabetic mothers were discovered to be risk factors for hypoglycemia.

This finding is consistent with another study conducted in Nigeria, which found that 28.3%^26^, and Ethiopia, 25%^8^. However, the finding is higher than the study conducted in Israel^22^, in Kenyatta National Hospital 14.7%^27^, in Tehran hospital 15.15%^28^, in India 21.2%^29^, and Uganda 2.2%^3^.

These differences may have been due to variations in the sampling size, study period, setting, and study design. Moreover, the level of the healthcare system regarding the detection and treatment of hypoglycemia is often different between developed and resource-limited countries or continents^30,31^. In this regard, the study was conducted in a level II neonatal intensive care unit with limited equipment or materials to support sick neonates and also limited opportunities for updated and advanced training to improve quality, which may increase the rate of neonatal hypoglycemia in the study area as compared with another study setting.

The odds of hypoglycemia among preterm neonates with hypothermia were 4.5 times higher as compared to preterm neonates without hypothermia. When a baby gets cold, he or she uses up more glycogen to keep warm, and then they must use their glucose stores to keep warm, which causes blood sugar to drop and the baby to become hypothermic, as well as hypoglycemic^32,33^. As a result, preventing and managing hypothermia through warm chains and skin-to-skin care is critical to avoid hypoglycemia and its associated complications^23,34^.

The odds of hypoglycemia among preterm neonates with birth asphyxia were 5.1 times higher as compared to a preterm with no birth asphyxia. Because anaerobic metabolism is used to maintain blood glucose concentrations in the neonatal period, birth asphyxia increases the risk of hyperinsulinism^35,36^. Prevention and treatment of birth asphyxia by trained and skilled healthcare providers is critical for every birth neonate, regardless of the cause, in order to avoid hypoglycemia and improve survival rates^37,38^.

The odds of hypoglycemia in preterm neonates with seizures were 4.7 times higher than in preterm neonates without seizures. This can result in altered glucose metabolism, the reduction of intracellular energy metabolites and phosphocreatine, and the accumulation of metabolic intermediates, such as lactate and adenosine, which result in neonatal hypoglycemia^39^. Thus, diagnosing and managing neonatal seizures is essential to improving the survival of neonates. The odds of hypoglycemia among preterm neonates born to diabetes mothers were 6.7 times higher as compared to preterm neonates born to non-diabetic mothers. Maternal diabetes is one of the major problems in neonates following a pregnancy complicated by diabetes. Another is hypoglycemia. Because glucose freely crosses the placenta, maternal hyperglycemia associated with GDM causes elevated glucose levels in the fetus, resulting in hyperinsulinism^40,41^. Thus, frequent monitoring and evaluation of glucose levels among delivered mothers is crucial worldwide in general and in the study area in particular to reduce the burden of hypoglycemia in neonates^42,43^.

Although this study attempted to explore hypoglycemia and its associated factors for the first time in the study, it has some limitations. Firstly, it was a cross-sectional study; it’s a snapshot, which is fronting for a chicken-egg dilemma, and secondly, the study was conducted in a single setting, Hence, risk validation and development model, randomized control trials, and/or cohort studies are recommended to better realize the detection rate of hypoglycemia and associated factors among neonates.

## Conclusion

The proportion of hypoglycemia and associated factors among preterm neonates admitted to the neonatal intensive care unit at Debre Tabor Comprehensive Specialized Hospital was found to be high. The associated factors for the occurrence of hypoglycemia were discovered to be neonates with hypothermia, birth asphyxia, seizure, and neonates born with a diabetes mother. Thus, recognizing and treating the above associated factors is essential to preventing, and controlling hypoglycemia.

## Data Availability

Data will be had upon request from the corresponding author.
